# Supplementary material for: Empirical methods for controlling false positives and estimating confidence in ChIP-Seq peaks
Source: BMC Bioinformatics. 2008 Dec 5;9:523. doi: 10.1186/1471-2105-9-523 (PMC2628906; doi:10.1186/1471-2105-9-523)
Supplement: Additional File 1 — A variety of html documents from the USeq web site detailing the available applications, their best usage, output file type descriptions, command line menus, etc. [file 1471-2105-9-523-S1.zip › USeqUserGuides/JavaDoc.zip!/JavaDoc/edu/utah/seq/analysis/PeakPicker.html]

PeakPicker


|  |  |  |
| --- | --- | --- |
| **Overview** \| **Package** \| **Class** \| **Use** \| **Tree** \| **Deprecated** \| **Index** \| **Help** | |  |
| **PREV CLASS**   **NEXT CLASS** | **FRAMES**    **NO FRAMES**     **All Classes** |  |
| SUMMARY: NESTED \| FIELD \| CONSTR \| METHOD | DETAIL: FIELD \| CONSTR \| METHOD |  |


---


## edu.utah.seq.analysis Class PeakPicker

```
java.lang.Object
  edu.utah.seq.analysis.PeakPicker
```

---

``` public class PeakPicker extends java.lang.Object ```

Use to find the peaks within EnrichedRegions

---

**Constructor Summary**

`PeakPicker(EnrichedRegion[] enrichedRegions,
java.io.File[] treatmentPointDirs,
java.io.File[] controlPointDirs,
int windowSize)`
  
          Picks the best peak based on rescanning each enriched region using the defined window size.


| **Method Summary** | |
| --- | --- |
| `static SmoothingWindow` | `findHighestPeak(SmoothingWindow[] sm, int scoreIndex)`             Find the window with the largest score. |
| `void` | `findPeaks()`             Finds the best peak within each loaded enriched region. |
| `void` | `pickPeaks()`             Main method of class. |
| `void` | `scoreTreatments()`             Used for window scanning treatment only datasets |
| `void` | `scoreTreatmentsControls()`             Used for window scanning with both treatment and control data. |

**Methods inherited from class java.lang.Object**

`equals, getClass, hashCode, notify, notifyAll, toString, wait, wait, wait`

**Constructor Detail**

### PeakPicker

```
public PeakPicker(EnrichedRegion[] enrichedRegions,
                  java.io.File[] treatmentPointDirs,
                  java.io.File[] controlPointDirs,
                  int windowSize)
```

:   Picks the best peak based on rescanning each enriched region using the defined window size.


**Method Detail**

### pickPeaks

```
public void pickPeaks()
```

:   Main method of class. This method will then re window scan each EnrichedRegion and set the subWindows and bestSubWindow.

---


### findPeaks

```
public void findPeaks()
```

:   Finds the best peak within each loaded enriched region. Call scoreTreatmentsControls() or scoreTreatments() first.

---


### findHighestPeak

```
public static SmoothingWindow findHighestPeak(SmoothingWindow[] sm,
                                              int scoreIndex)
```

:   Find the window with the largest score. If multiple windows have the same best score then the start of the first and end
    of the last are used to create a new window and are returned.

---


### scoreTreatmentsControls

```
public void scoreTreatmentsControls()
```

:   Used for window scanning with both treatment and control data.

---


### scoreTreatments

```
public void scoreTreatments()
```

:   Used for window scanning treatment only datasets


---


|  |  |  |
| --- | --- | --- |
| **Overview** \| **Package** \| **Class** \| **Use** \| **Tree** \| **Deprecated** \| **Index** \| **Help** | |  |
| **PREV CLASS**   **NEXT CLASS** | **FRAMES**    **NO FRAMES**     **All Classes** |  |
| SUMMARY: NESTED \| FIELD \| CONSTR \| METHOD | DETAIL: FIELD \| CONSTR \| METHOD |  |


---
